# Insights Into Older Adult Patient Concerns Around the Caregiver Proxy Portal Use: Qualitative Interview Study

**DOI:** 10.2196/10524

**Published:** 2018-11-02

**Authors:** Celine Latulipe, Sara A Quandt, Kathryn Altizer Melius, Alain Bertoni, David P Miller Jr, Douglas Smith, Thomas A Arcury

**Affiliations:** 1 Department of Software and Information Systems University of North Carolina at Charlotte Charlotte, NC United States; 2 Department of Epidemiology and Prevention, Division of Public Health Sciences Wake Forest School of Medicine Winston-Salem, NC United States; 3 Department of Epidemiology and Prevention, Division of Public Health Sciences, Department of Internal Medicine Wake Forest School of Medicine Winston-Salem, NC United States; 4 Department of Internal Medicine Wake Forest School of Medicine Winston-Salem, NC United States; 5 Greene County Health Care, Inc Snow Hill, NC United States; 6 Department of Family and Community Medicine Wake Forest School of Medicine Winston-Salem, NC United States

**Keywords:** caregivers, patient portals, proxy, proxy portal access, proxy portal accounts

## Abstract

**Background:**

Electronic patient portals have become common and offer many potential benefits for patients’ self-management of health care. These benefits could be especially important for older adult patients dealing with significant chronic illness, many of whom have caregivers, such as a spouse, adult child, or other family member or friend, who help with health care management. Patient portals commonly contain large amounts of personal information, including diagnoses, health histories, medications, specialist appointments, lab results, and billing and insurance information. Some health care systems provide proxy accounts for caregivers to access a portal on behalf of a patient. It is not well known how much and in what way caregivers are using patient portals on behalf of patients and whether patients see any information disclosure risks associated with such access.

**Objective:**

The objective of this study was to examine how older adult patients perceive the benefits and risks of proxy patient portal access by their caregivers.

**Methods:**

We conducted semistructured interviews with 10 older adult patients with chronic illness. We asked them about their relationship with their caregivers, their use of their patient portal, their caregiver’s use of the portal, and their perceptions about the benefits and risks of their caregiver’s use of the portals. We also asked them about their comfort level with caregivers having access to information about a hypothetical diagnosis of a stigmatized condition. Two investigators conducted a thematic analysis of the qualitative data.

**Results:**

All patients identified caregivers. Some had given caregivers access to their portals, in all cases by sharing log-in credentials, rather than by setting up an official proxy account. Patients generally saw benefits in their caregivers having access to the information and functions provided by the portal. Patients generally reported that they would be uncomfortable with caregivers learning of stigmatized conditions and also with caregivers (except spouses) accessing financial billing information.

**Conclusions:**

Patients share their electronic patient portal credentials with caregivers to receive the benefits of those caregivers having access to important medical information but are unaware of all the information those caregivers can access. Better portal design could alleviate these unwanted information disclosures.

## Introduction

### Background

The introduction of electronic patient portals over the past decade has the potential to offer many benefits to patients, such as faster and more direct access to health information and test results and the ability to easily renew prescriptions, make appointments, and communicate with health care providers. However, these benefits may not accrue uniformly across patient populations, and research has shown that patient portal adoption is lower among older adults, who typically have more chronic health problems and increased needs for health services [1].

Many older adults have limited ability and motivation to use electronic patient portals [2]. However, their caregivers (such as adult children) may be able to leverage the benefits of such portals on their behalf. This usage of patient portal accounts by a caregiver is referred to as *proxy portal use*. Some portal systems allow patients to provide a caregiver with access to their portal by setting up a proxy account. While health care systems that offer proxy access may encourage patients to set up proxy accounts for their caregivers, patients may simply share their portal credentials (username and password) with their caregivers.

The objective of our research was to gain insight into how older adult patients with chronic illness think about caregiver access to information available on patient portals. We have presented a qualitative interview study with lower-income, older adult patients in an urban area in the United States. We interviewed patients about their caregiver relationship, their own use of their patient portal, their caregiver’s use of their patient portal, and their comfort levels in sharing all the information and functionality that a standard patient portal provides. We discuss our findings and provide a set of preliminary design guidelines for patient portal designers who wish to support this proxy portal use.

### Barriers to Adoption of Patient Portals

Electronic patient portals can be viewed as an information and communication technology that supports “aging in place” because it allows older adults to access tools and information to manage their chronic illness from their own homes. Connelly et al have found that the evaluation of information and communication technologies for aging in place requires multiple methods because of the wide variety of nuanced contexts in which older adults live [3].

Lyles et al showed that lack of technical support and the fear of losing the doctor-patient relationship were barriers to the adoption of patient portals for African American and Latino patients [4,5]. Similarly, Ancker et al found that the odds of being given an access code for a patient portal were higher for patients who were young, English speakers, white, insured, and female [6]. Patients with lower socioeconomic status were less likely to make use of patient portals [7].

Health literacy may be another significant factor impacting the adoption of patient portals, as it is correlated with higher perceived ease of use and usefulness of health information technologies [8]. Functional impairment may negatively impact the ability of older adults to use patient portals [9]. Given that functional impairment tends to be lower with younger populations [9] and that health literacy tends to be higher among younger populations [10], younger caregivers may be better equipped to use patient portals than some older patients.

### Caregivers and Information Sharing

Caregivers (often family members) play an important role in assisting older adults living with chronic medical conditions. According to a 2013 Pew Research report, 36% of US adults acted as caregiver to an adult relative or friend [11]. Some recent research suggests expanding the concept of “personal health informatics” to “family health informatics” [12]. Pang et al have noted the need to design for patient privacy while sharing information with relatives [13]. The needs of informal caregivers, such as their need for social support, recognition as caregivers, and communication with other stakeholders, are complex [14-18]. Bosch and Kanis noted the importance of making caregivers better-informed through access to systems such as patient portals [19].

Recent work looking into chronic illness patients with spousal caregivers demonstrates that it is important to support situations in which the values between patient and caregiver are in tune, as well as situations in which these values conflict [20]. While that work targeted spousal caregivers, similar patterns of conflicting values may emerge with other types of caregivers such as adult children, siblings or close friends, and neighbors. Designing to handle conflicting values around sharing and privacy between patient and caregiver becomes a critical component when looking at proxy access to patient portals.

### Proxy Portal Accounts

Most electronic patient portal systems have some mechanism for the provision of proxy accounts. This is common for parents of young children, where the parents get automatic proxy access to their children’s portals. However, only some patient portal systems allow proxy accounts to be established for adult patients. Health care systems that do not offer proxy accounts for adult patients may recommend that adults wishing to share their patient portal information share their log-in credentials with their caregiver. Sarkar and Bates argued that current health care systems do not adequately engage caregivers and noted, “…although the Office of the National Coordinator acknowledges the importance of caregivers and family, broadly adopted standards for caregiver access to patient portals are not available” [21].

Caine et al have investigated patient attitudes toward the sharing of their electronic health records within medical systems [22]. They found that patients were unaware of how much information was in their electronic health records and wanted much more fine-grained control over who could access this information. In addition, they wanted to know when their information was accessed and by whom. While their study did not specifically address issues of patient portals and caregiver access, the issues they discussed are relevant to the patient portal context.

In a survey of patients across all age groups, Wolff et al found that patients share portal access with caregivers not only for information sharing purposes and emergency reasons but also because they need technical help [23]. In a focus group study of adults aged >75 years, participants raised concerns about autonomy and control, and the authors reported that it would be difficult to create a single model of access control for proxy accounts that would be suitable for all patient-caregiver dyads [24]. In addition, Wolff et al conducted a scan of 20 large health systems in the United States to understand how many offer proxy portal accounts for adult caregivers and noted that while many do offer such accounts, only some health systems advertise the availability of proxy accounts on their websites [25].

### Health Information Security

How health information is shared within systems has been a subject of study, particularly when patient charts include information about stigmatized conditions such as mental illness, substance abuse, or sexual health [26]. In addition, Caine and Hanania and Schwartz et al showed that patients want granular control over which health care stakeholders see what parts of their medical data [27,28]. We are unaware of any studies specifically examining how patients deal with such information when sharing patient portal access with a caregiver, who may have a proxy account or who may have the patients’ portal password.

There are 2 interrelated issues with password sharing. Individuals frequently share their passwords with friends, coworkers, and relatives, allowing them to access a system account using the account holder’s credentials [29,30]. In addition, individuals often engage in cross-system password sharing, in which they use the same password (or a similar password) across many systems [31,32]. The sharing of passwords with others creates a significant information disclosure risk if the individual owning the credentials has also used the same password across multiple systems, such as a patient portal, Web-based banking, and social networking.

One approach to improving privacy in the sharing of health information is the “break-glass” access control protocol [33-35]. This idea is based on the metaphor of the need to break the glass around a fire alarm. This can be applied to health care information because many people want to keep their health information private, but in the case of a life-threatening medical emergency would want caregivers to have access to that health information. When this approach is applied to health information systems, any emergency that satisfies the “break the glass” criteria not only allows specified people to access the information but also creates an audit trail so that patients and providers can see that the information has been shared, when it was shared, and with whom. To the best of our knowledge, this approach has not been used for managing access to patient portals for patient caregivers.

## Methods

### Research Project

This research fits within a larger longitudinal research project investigating older adult patients and caregiver usage of electronic patient portals [2,36]. In that project, a set of patients and caregivers have been interviewed periodically over a period of 2 years to understand the patient portal adoption and usage. The patient portal at the academic medical center that is the home for our research team does offer proxy accounts for caregivers of adult patients. Creating this account requires completing and submitting paper forms.

For this particular study into patient perceptions around caregiver access, we began by first doing a walkthrough of the patient portal system. We analyzed the functionality available in this patient portal and compared that with the functionality in other portal systems to generate a set of common features. We then developed an interview study to more deeply understand how older adults perceive caregiver’s proxy portal access within the context of this standard feature set.

### Participants

The recruitment objective was to engage patients who were racially diverse and representative of the population of older, low-income adults in the area surrounding the academic medical center. Participants were selected from among individuals who had participated in the larger longitudinal survey interview study of patient portal utilization [2,36]. Low-income older adults were the focus of the larger study because this population has relatively low use of patient portals and digital technology, in general, and a goal was to determine leverage points for improving the patient portal utilization in this segment of the population.

For the larger study, 120 participants were recruited from patients receiving care from a clinic in the academic medical center that serves predominantly Medicaid and noninsured patients. Participants were not asked to meet low-income criteria as individuals. These participants were community-dwelling adults aged ≥55 years, who were being treated for a chronic disease (diabetes, hypertension, dyslipidemia, or cardiovascular disease), spoke English or Spanish, and were in sufficiently good health to give informed consent and complete a lengthy fixed response survey interview. Recruitment for the larger study included a 3-step process: (1) clinic staff and physicians generated a list of patients who met the inclusion criteria; (2) we sent recruitment letters to a randomly generated list of these patients; and (3) we made follow-up phone calls to describe the study and schedule interviews with those who were sent the letters. The overall study had a refusal rate of 49.2%.

Of 70 academic medical center patients who had a caregiver when they completed the survey interview component and who remained in sufficiently good health to continue participation, we recruited 10 to complete the semistructured interviews (age range, 60-71 years; 5 male, 5 females; 7 African American, and 3 white individuals; see Table 1). Participants were contacted individually until 10 agreed to complete the interview.

**Table 1:** The summary of participants, portal use, caregiver relations, caregiver portal use, and the presence of stigmatized health condition.

Participant identifier	Sex	Race	Uses portal	Caregiver relation	Caregiver uses portal	2^nd^ caregiver relation	2^nd^ caregiver uses portal	Stigmatized health condition^a^
P1	Female	African American	Yes	Child	Yes	N/A^b^	N/A	N/A
P2	Male	African American	Yes	Friend	No	N/A	N/A	HIV
P3	Female	African American	Yes	Children	Yes	Home health assistant	Yes	MH^c^
P4	Male	White	No	Spouse	Yes	N/A	N/A	N/A
P5	Male	African American	Yes	Brother	No	Home health assistant	No	N/A
P6	Male	African American	Yes	Spouse	No	N/A	N/A	MH
P7	Male	White	Yes	Sister	Yes	Neighbor	No	N/A
P8	Female	White	Yes	Spouse	No	N/A	N/A	MH
P9	Female	African American	No	Niece	No	N/A	N/A	MH
P10	Female	African American	No	Child	No	N/A	N/A	N/A

^a^All patients suffered from at least one chronic condition such as high blood pressure, arthritis, or heart disease.

^b^N/A: not applicable.

^c^MH: mental health.

### Interview Structure

Data collection was completed between June and August 2017 by 2 trained interviewers. Interviewers met participants in their homes. The interviews typically lasted between 30 and 60 minutes and were audiorecorded. The interviewers explained the study and obtained signed consent. Participants received a US $20 incentive for completing the interview. The interview study was approved by the academic medical center’s Institutional Review Board.

The semistructured interview covered patient’s background, caregiver relationship, patient’s portal use, and caregiver’s use of the portal (see Multimedia Appendix 1). We defined caregiver as “someone with whom you share your health information, and who helps you with your health care.” We asked all patients about how secure they considered their health information on the portal, and whether they considered it more or less secure than social networking sites and Web-based banking. The interview then specifically investigated the patients’ comfort level with caregiver access to the portal in the hypothetical situation of a stigmatized illness, as well as the comfort level with the caregiver accessing information such as past medical records and billing or insurance data. This part of the interview included laminated screenshots of the patient portal with notional data (such as screens showing provider messaging, prescription renewal, test results, visit summaries, appointment scheduling, and billing), which were used as visual prompts to remind participants of the various features being discussed and the kinds of information available on various screens of the portal. We specifically asked participants about whether they would be concerned with their caregivers seeing financial billing information on the portal. We asked this to ascertain whether participants were aware of this information being on the portal and how they felt about sharing such information.

### Data Analysis

Transcripts from the audiorecorded interviews were completed by a professional service and then edited for accuracy. Data analysis was conducted throughout the process, with the team performing ongoing reflection on interview transcripts as they became available. As the researchers reviewed the transcript narratives, they met periodically to discuss the themes, patterns, and issues they found in the data [37]. The research team created an initial coding dictionary based on these discussions. The codes included in the dictionary reflect *a priori* themes reflected in the interview guide and themes that arose from the review of the data. Two of the interviews were coded and reviewed by 4 researchers who then refined the coding dictionary. All interview transcripts were then coded in a 2-step process. Each transcript was coded by one research team member and then checked for coding accuracy by a second team member. All of the investigators discussed the interpretation of the themes and patterns, and agreed on the final presentation.

## Results

### Principal Results

In total, 7 patients had used the patient portal provided by the academic medical center, and 4 of them had allowed one or more of their caregivers to access their patient portal (see Table 1). Given the small sample size, these numbers are not meant to suggest typical levels of portal usage by patients or caregivers but suggest that there is some proportion of older adult patients that use the portal and some portion that do allow their caregivers to access their portals on their behalf.

### Caregiver Relations and Tasks

All participants identified at least one caregiver. Most participants identified a close relative, such as a spouse, sibling, or adult child, as their primary caregiver. Caregivers helped with both health-related and household tasks such as cleaning and cooking. The health care–related tasks that caregivers engaged in included getting patients to appointments, reminding them of medications, helping with diet and exercise, and communicating with doctors.

Eight participants had given their caregiver Health Insurance Portability and Accountability Act of 1996 (HIPAA) authorization by signing an authorization document. Two participants were unfamiliar with HIPAA and were unsure whether or not they had granted anyone such authorization. Three participants identified multiple caregivers, and in these cases, there was a split of responsibilities with a local friend or paid assistant helping with day-to-day care, and a family member acting as a caregiver from a distance.

### Patient Portal Use

Overall, 7 of 10 participants used their patient portal, though their perceptions of it and usage levels varied. P3 reported using her portal 4 times per week and mentioned using the portal for appointment tracking. P1 reported using the portal but explained that she did not like it at all, mainly because her health care providers had not responded to messages through the portal. Other participants had logged into the portal occasionally, and 2 of the participants had forgotten their portal passwords and had not been able to log in recently. Participants reported using the portal to monitor appointment schedules, message with doctors, and look at test results. None of the participants reported using the portals to look at billing or make payments.

### Security Concerns

All participants remarked that they expected the information to be seen by health care staff only and that information on the portal was kept more private than information on Facebook. In comparing the portal to Web-based banking, a number of participants felt they could not comment because they did not do Web-based banking, while others felt the portal was equally secure or more secure than Web-based banking. Most participants thought it was very unlikely that someone could hack into their portal and steal their health information. Participant responses demonstrated that they do not consider their medical information to be of high value or interest and are therefore not very concerned about information security issues when using the portal.

### Caregiver Portal Use

Of 10 participants, 4 had given their passwords to one or more caregivers so that the caregiver could access the portal. P1 talked about how she had set her portal password to something easy to remember and shared it with her daughter, who mostly checks it for appointments. In asking about who has access to her portal, P3 noted that multiple children and her home health care assistant all have access, but only use it on an as-needed basis. P5 has 2 caregivers—a daily home health care assistant and a brother with whom he shares his health care issues. His brother, who has HIPAA authorization and power of attorney, can log into his patient portal but has not done so to his knowledge. P5 has not given his home health care assistant access to his patient portal, despite noting that they are “good friends.” In addition, P7 has 2 caregivers, a local neighbor and a sister who lives in another state. His sister has access to the portal and used it to follow appointments as P7 went through cancer treatment. However, P7 has not given his neighbor caregiver access. The neighbor takes him to the hospital in medical emergencies and helps him when he is not well, and P7 says if it became important or useful, he would consider giving her access to the portal.

### Thematic Analysis

A number of themes emerged around comfort level with caregiver portal access. Most participants, even those who were not on the portal or who had not granted caregivers access to the portal, saw benefit in their caregiver being able to access the portal. Although 2 of 10 patients interviewed (P6 and P8) used their portal themselves, they had caregivers who were not internet users and would be unable to use the patient portal.

### Health Literacy Assistance

One of the main reasons participants felt caregiver access was beneficial was in the caregiver’s ability to help them understand the information on the portal, such as doctor’s messages and test results. For example, P3 described how her caregivers help her understand the information that is there.

I don’t understand all this, all these abbreviations. So she [health assistant] opened it up, she and my daughter-in-law, and they were reading and letting me know that my red blood cell count was very low and my white blood cells were my hemoglobin was out of whack, my TSH levels were out of whack.

Similarly, P8 mentioned a close friend who works in the medical field and could act in the role of caregiver (though does not currently). She noted how helpful it could be to have this friend access her portal, saying “she may understand some of the stuff better than I do.”

### Caregivers as Communication Gatekeepers

In multiple instances, caregivers helped to keep the rest of the family informed or helped to explain difficult medical situations to family members. For example, P3 who gives her daughter-in-law portal access, noted the following:

My son is one of those people that he can’t take what’s happening, so I explain it to her [daughter-in-law]. She’s in the medical field as well, and she’s a CNA [Certified Nursing Assistant] at [clinic] and she and her best friend [name] is a registered nurse, they get together and explain to him what’s going on with me.

This quote demonstrates both the benefit of caregivers with medical knowledge having portal access and those caregivers using that information to communicate the situation to other family members. P7 described the benefits of his sister having portal access.

She, sort of keeps the other family members informed, so it serves a purpose there, too.

### Stigmatized Health Issues

The portal in use at the academic medical center is full featured and includes full medical records and past diagnoses. When asked about the security of information on the portal, P1 who is a regular portal user and was familiar with the amount of information available noted the following:

Now, probably if I were HIV+ and was trying to get a job, I might be a little more sensitive about things like that. But I don’t know. I’m not real worried about it.

This comment demonstrated that the concerns about the privacy of information on the patient portal may be moderated by the presence or absence of stigmatized health issues (both from the perspective of the portal being hacked and the perspective of the portal being accessed by caregivers). P2 had an HIV+ diagnosis, and he expressed reticence about sharing his patient portal with his caregiver friend. This participant noted that he had not shared his HIV+ status with his caregiver, although he admitted that he would be okay with her finding out about his status through the portal if there was an emergency and she needed access to help him.

P5, when asked about caregivers accessing information about a hypothetical HIV diagnosis, responded as follows:

You don’t want everybody to know, but then the ones that are close to you that are actually gonna have to be the ones there for you, you would have to let them know.

This quote highlights that there is a tension between needing care help and feeling embarrassed about such a stigmatized condition.

P7 raised a number of concerns about caregiver portal access. One concern was that his neighbor caregiver, who does not drink alcohol, would see that he drinks alcohol if she had access to the portal. This demonstrates that having caregivers who are slightly more distant in relationship can cause tensions in considering sharing access to a patient portal because lifestyle information is often captured and recorded as part of routine health checkups. In addition, P7 did not like the idea of his caregivers learning about a hypothetical mental health diagnosis. He also expressed a concern about a hypothetical diagnosis of HIV.

I don’t think I’d want them to know. No, I don’t think so. I think that would be considered sort of private, … I mean I hope that never happens, but I wouldn’t feel real comfortable.

### Emergency Access

Several patients discussed the benefits of caregivers being able to access the portal in case of an emergency. While patients who had granted portal access to their caregivers saw benefits of this access in cases of emergency, this was also true for participants who had *not* granted portal access to their caregivers. This was clear in the case of P2, the participant who had an HIV+ status, but had not shared that status with his caregiver. He admitted that he would be okay with his friend caregiver accessing his portal in case of emergency, but his comment expresses a high level of reticence, even about emergency access: “…if it had to be, so be it. If it came down to it.” The finality of this comment suggests that giving her access would definitely be a last resort, only if he really needed help from her. P7 has a sister living out of town who serves as a distant caregiver and a neighbor who serves as a close-by caregiver. This was the participant mentioned previously who felt that he would not want his neighbor to have access to the portal because she might be able to see that he drinks alcohol. But he expressed that in an emergency, he could see the benefits of her being able to access the portal and communicate with his out-of-town family.

### Billing Privacy

Most participants, even those who were regular users of the portal, were unaware that billing information was available on the portal. Some participants who had close family members as caregivers were not concerned with those caregivers seeing billing information. P4 explained her openness in this area:

That would be fine too because, if something happens to us, we’re older, they’re gonna be responsible for that.

P9 noted that she would not want her niece caregiver to see the balance in her bank account, but she would be okay with her niece seeing the medical bills on the patient portal.

Some participants had definite concerns about their caregivers accessing their portal and seeing billing information, and this tended to vary with the relationship between the patient and the caregiver. For example, P5 responded that he would be comfortable with his brother seeing billing information but not his home health care assistant. P8, who has a husband caregiver who does not use the portal at all, spoke about possibly giving her best friend portal access and described how she would trust her friend with health information, making appointments, and renewing prescriptions, but, “Well, I’d rather keep the billing stuff private… I’ve just always been, my finances are my business.”

P7 was not comfortable with anyone having access to his billing information. He did not realize that there was billing information on the portal, and in thinking about his sister and neighbor caregivers, noted “I would rather they not see that…an invasion of my private life, I guess.” These comments show that feelings about caregivers seeing medical billing information are quite varied, with some participants feeling quite uncomfortable about such information being disclosed to caregivers through the portal.

## Discussion

### Principal Findings

Participants in this study typically shared their current health information with their caregivers, whether through the portal or other means of communication. The only exception to this rule was when there was a stigmatized condition, such as in the example of the participant who is HIV+ and had not shared that status with his caregiver, but would be okay if she found out the status if she needed to, in case of emergency. This situation is a perfect example of where the “break-glass” paradigm of access control [33] could be applied to good effect. That 8 of 10 participants had given their caregivers formal HIPAA authorization reflects their willingness to share current health information. The other 2 participants indicated they were not familiar with HIPAA and were unsure if they had granted anyone authorization. With HIPAA authorization, caregivers can receive participant health information directly from health care providers.

We found that older adult patients see benefits in having their caregivers access the patient portals, though this was more likely when the caregiver was a family member. In this study, 4 of the 10 participants gave caregivers access by sharing their log-in credentials. The fact that none of these participants set up official proxy accounts for their caregivers suggests that they either did not know those accounts were available or they considered the process to get those accounts too burdensome. Regardless of the reason for password sharing, the practice is concerning, especially given research that shows people use the same or similar passwords across multiple systems [30,31]. Older patients who share their portal password with a caregiver may also be inadvertently giving that caregiver access to their bank account or email. However, this practice is an easy way for older patients to share their information with their caregivers and is likely to continue regardless of how easy proxy account setup becomes.

We observed that some patients have concerns about sharing information about stigmatized medical issues (such as mental health conditions or infections that could have been transmitted through sexual activity), though it tends to be hard for patients to consider these hypotheticals. The hypotheticals that caused the most privacy concerns were related to mental illness and sexually transmitted infections.

Furthermore, we noted significant concern from some of our participants regarding the billing information that is available in the portal being accessible to caregivers. While people tend to be quite private about their personal finances, this issue did not pose a concern when the caregiver was a spouse.

### Design Considerations

While patient portal systems allow for the setting up of proxy accounts, those accounts provide proxy users with access to everything that the patient can access. The following considerations are based on the idea that existing proxy accounts could be modified to allow the benefits of caregiver access, while addressing the common privacy concerns noted by our interview participants. These considerations should be taken as starting points for further research and discussion, given that our findings are based on a small sample of 10 patient interviews.

- Promote the use of proxy accounts by allowing easy setting up of proxies online, using simple, clear language.
- Provide a simple checklist of access controls, with screenshots, to help patients decide what information or functionality to grant the caregiver.  
- Provide a default proxy account configuration that includes access to most information and functions, but requires an opt-in for the complete medical record, billing, and insurance information.

These guidelines will only be useful if patients actually set up official proxy accounts for their caregivers. Some patients will likely continue to share their portal credentials with caregivers, and some health care systems do not offer proxy accounts for caregivers, which means password sharing is the only way for caregivers to access a patient’s portal. Hence, we offer the following design guidelines for systems to help mitigate issues when passwords are shared with caregivers:  

- Remind users when creating or changing passwords on the portal that they should choose unique passwords that are different from passwords on other important systems such as email and Web-based banking.  
- Provide a “break-glass” mechanism that allows patients to specify who can be given access to the portal in case of emergency. Then, ensure that the system logs that access and provides clear alerts and log-in history on the portal so that the patient is made aware when someone has used the emergency “break-glass” mechanism.
- Ask users to identify themselves when logging into a portal. For example, after logging into a portal, the system could prompt the user to define themselves as either the “patient” or a “caregiver.” If the user chooses caregiver, the system could ask their name and relationship. This could then be added to all of the logs and communications inside the portal so that the patient and caregiver would be separately identified. This could help the patient be able to see when their caregiver logged in and monitor for abuse of the system. Moreover, this would help health care providers know who they are communicating with through the portal.

One issue with the last guideline of having an identification step after log-in is that it could actually be seen as condoning or encouraging the sharing of portal credentials. One way to ensure that patients and caregivers do not view this as condoning the sharing of passwords is to respond when a caregiver self-identifies by asking them to talk to their patient about setting up proxy access and by sending a message to the patient that encourages them to set up a proxy account for that caregiver. In this way, the practice is allowed, but the system also nudges users toward a more secure mode of interaction with the system. This only makes sense if the patient portal system provides adult proxy accounts, and it is currently unclear how many portal systems in the United States actually provide this functionality.

### Limitations

This qualitative study has a small number of participants, which is a limitation to the generalizability of the results. Further studies with larger populations are needed to understand the prevalence of these caregiver portal access issues. Similarly, the elderly, low-income nature of our population limits the generalizability of our results. Younger and wealthier patients may have different concerns about caregiver portal access.

### Conclusions

We have presented the results of a qualitative study with 10 older, low-income adults who receive outpatient primary care through a university medical center in a small city in the United States. We have investigated how these older patients share health information with their caregivers, and how these patients feel about sharing electronic patient portal access with caregivers. While 2 previous studies have investigated patient attitudes about proxy portal use, ours is the first study to frame these attitudes around the full set of standard portal features and consider both the security and privacy concerns that may come into play. Our results suggest that patients typically share their log-in credentials with caregivers rather than setting up official proxy accounts. Regardless of the access mechanism, this proxy portal access provides no granularity of control over the information shared through the portal, and patients express some discomfort with the sharing of data around stigmatized illnesses and financial obligations. We suggest some guidelines to improve both official proxy portal accounts and standard portal accounts, to allow all stakeholders to reap the benefits of caregiver proxy portal use, without incurring inadvertent information disclosure risks or other security breaches.

## Appendix

Multimedia Appendix 1 Interview guide.

## Abbreviations

HIPAA: Health Insurance Portability and Accountability Act of 1996
