# Supplementary material for: Insights Into Older Adult Patient Concerns Around the Caregiver Proxy Portal Use: Qualitative Interview Study
Source: J Med Internet Res. 2018 Nov 2;20(11):e10524. doi: 10.2196/10524 (PMC6240158; doi:10.2196/10524)
Supplement: Multimedia Appendix 1 [file jmir_v20i11e10524_app1.pdf]

Maximizing the Impact of ePHIM in Low Income, Multiethnic Populations

Component 3: Patient Interview Guide for Proxy Use of Portals

0: Introduction

Today I would like to speak with you about the person who you share your health information with, and who helps you with your health care. We will call this person a "Care Helper." They can also be called a "Care Partner," or a "Caregiver."

> Many people have someone who helps them with activities and questions about their health. These activities and questions include simple things, like reminding you to take your prescription and about an upcoming doctor's appointment, or finding information about something the doctor has told you; they can include more substantial assistance, like taking you to an appointment, helping you take your medicine, and helping you exercise; and they can include personal assistance, like helping you get dressed and bathe. Those who help you can be your spouse, brothers or sisters, children, or friends. The person who helps you may live with you, but they can also live in another house. They might even live in another town or city and help you by phone or the internet.

I would also like to talk about how you feel about your Care Helper having access to your patient portal.

1: Warm-up

We have interviewed you before, so we know a little about you. But just so I can be sure . . .

Tell me about yourself – your age, where you are from, where you went to school, what kinds of jobs you have had.

Tell me about your current health status.

2: Care Helper / Care Partner / Caregivers

Now, let's talk about your care helper (care partner, caregiver).

Remember, a care helper is someone who helps your with your health care – anything from talking to you about your health to giving you direct care.

Can you tell me who your care helpers are?

> Who helps you with your health care by doing things like going with you to doctor's appointments, picking up your prescriptions, making sure you are taking the right medicines, etc?
> Can you tell me about all the people who do that kind of thing for you?"

[if more than one] Is one of the people you listed your primary care helper, the person who is most involved in helping you manage your health and getting health care?

Tell me about the person who helps you most with your health and getting health care. This person may only help you a little.

What is their name?
How is this person related to you? Spouse, partner, child, friend?

What are the ways in which [care helper] helps you?

Is [care helper] someone with whom you can talk about your health and your health care?
Does [care helper] remind you to take your medicine … about your diet … about exercise?
Does [care helper] help you get your prescriptions … help you make appointments … take you to appointments?
Does [care helper] help take care of you after surgery and other procedures?
What aspects of your health do you <u>not</u> share with [care helper]?

Tell me about any other people who help you with your health and getting health care?

[If no one else mentioned] They could include family members or close friends who help every once in a while, such as during a bad illness.

How do they help you with your health and getting health care?

What are ways [care helper] can get information about your health?

You talk to them about your health?
They see your insurance statements? They see your medical bills?
They are with you when you have an appointment with a doctor or other health care provider?

[May discuss portal here.]

What are the ways [care helper] communicates with your doctor or your doctor's office?

They are with you when you have an appointment?
They e-mail your doctor's office?

Some people have a hard time understanding or remembering everything their doctors tell them. Are there ways that [care helper] helps you understand or remember what your doctors tell you?

Have you provided [care helper] with "HIPAA authorization" to have access to your health information?

What kinds of health care information do you …

Share with [care helper]?
Want to share with [care helper]?
Don't want to share with [care helper]?

3: Patient Portal Privacy

Are you familiar with what a "patient portal" is? For people who receive health care from a medical practice affiliated with BLINDED FOR REVIEW, the patient portal is called "BLINDED FOR REVIEW." BLINDED FOR REVIEW allows patients to see their health records, make appointments, get prescription refills, and send questions to their doctors through the internet.

I want to ask you about privacy and your health information.

Do you feel that the health information stored about you on your patient portal is kept private?

Who do you think has access to the information on your portal?

Is your portal information any more or less private than Facebook? Or online banking?

How likely do you think it is that someone can hack in to the portal and steal your health information?

4: Care Helper Patient Portal Access

Did you know that [care helper] <u>can</u> be given access with your permission to your BLINDED FOR REVIEW patient portal?

Has [care helper] ever logged into your patient portal to help you access health care?

[if yes…]

Tell me about [care helper] logging into your patient portal. Like . . .

- How often has [care helper] logged on to your patient portal?
- What do they do on your patient portal?
- When [care helper] accessed your patient portal, was it with you present?
- Helping you use the computer to find information the patient portal? (Why, why not?)
- Helping you to understand the health information that is on the patient portal? (Why not?)

How do you feel about [care helper] using your patient portal? What concerns do you have, if any?

What are the potential benefits to you of [care helper] using your patient portal?

[if no…]

Why do you think [care helper] hasn't logged in to your patient portal?

How would you feel about [care helper] using your patient portal? What concerns do you have, if any?

What do you think might be the benefits to you if [care helper] used your patient portal?

How would you feel about having [care helper] being able to see and read all the information the doctor tells you about at your medical appointment?

What concerns do you have?

How about contact between your doctor and your [care helper]? How would you feel about them being able to communicate with each other about your health issues – even if you aren't present or don't know they are communicating?

Now, let's talk about some different health conditions and how those conditions might influence your willingness to share your patient portal information with different types of care partners.

If you had [INSERT CONDITION], how would you feel about letting [INSERT CARE PARTNER] have access to your patient portal?

If you had [INSERT CONDITION] and [INSERT CARE PARTNER] was your care partner, how comfortable would you be if [INSERT CARE PARTNER] had access to your patient portal?

	Spouse/Significant Other	Child	Sibling (sister or brother)	Neighbor	In-Home Health Aid
High Blood Pressure					
Heart Disease					
Diabetes					
Incontinence					

Women’s Health Issues					
Men’s Health Issues					
Cancer					
Cognitive Impairment (e.g., dementia)					
Mental Illness (e.g., depression or anxiety)					
More Serious Mental Illness (e.g., bipolar disorder or schizophrenia)					
Alcohol and Substance Abuse Related Issues (e.g., alcoholism; cirrhosis)					
HIV or other Sexually Transmitted Diseases					

What additional information would you not share?

What kinds of health information would you want to be confidential?

5: Detailed Comfort Level - Matrix of Access

In this section of the interview, I’m going to dig into the details to see what parts of the patient portal you would be okay with [care helper] seeing. I’m going to show you some pictures of the patient portal at BLINDED FOR REVIEW, but they are from a fictitious patient, not your portal. So these pictures are ‘made up’. But, I’d like you to imagine what your portal might look like when you see these pictures, okay?

[Repeat for each dimension in table below]

Here is a picture of the BLINDED FOR REVIEW portal showing where you can <see appointments>.

How comfortable would you be with [care helper] being able to [see appointments]”. Your responses can range from Very comfortable, somewhat comfortable, unsure, somewhat uncomfortable, or very uncomfortable.

> Can you tell why you feel that way?
>> What makes you feel comfortable about . . .
>> What makes you feel uncomfortable about . . .

Dimension	Very comfortable	Somewhat comfortable	Unsure	Somewhat uncomfortable	Very uncomfortable
4a. See appointments					
4b. Make appointments					
4c. See messages from doctor/healthcare staff					
4d. Send messages to doctor/healthcare staff					
4e. Renew prescriptions					
4f. See doctor visit summaries					
4g. See hospital admission summaries					
4h. See billing account					
4i. See insurance information					
4j. See medical record, which includes conditions, test results, health summaries, medications, medical history and immunizations					

Finally, would you be comfortable with [care helper] having partial access to your patient portal, if you could control the types of information they would have access to? So, for example, if you could allow [care helper] to see appointments and renew prescriptions, but not see test results. How would having that kind of control over <primary caregiver's> access to your portal make you feel?

Note to Interviewer: if patient really does have multiple caregivers (i.e., both husband and daughter act as caregivers), repeat Parts 2-4 for other caregiver. Not necessary for a tertiary caregiver such as a neighbor who just occasionally picks up prescriptions for patient.
